# Reduced Presence of SARS-CoV-2 microRNA-like Small RNA in the Serum of Patients with Post-Acute Sequelae SARS-CoV-2 Infection

**DOI:** 10.3390/microorganisms13010126

**Published:** 2025-01-09

**Authors:** Maria Alfreda Stincarelli, Isabella Abbate, Giulia Matusali, Michele Tanturli, Marta Camici, Rosaria Arvia, Elisabetta Lazzari, Eleonora Cimini, Alessandra Vergori, Fabrizio Maggi, Simone Giannecchini

**Affiliations:** 1 Department of Experimental and Clinical Medicine, University of Florence, Viale Morgagni 48, 50134 Florence, Italy; mariastincarelli@gmail.com (M.A.S.); rosaria.arvia@unifi.it (R.A.); 2 Laboratory of Virology and Biosafety Laboratories, National Institute for Infectious Diseases Lazzaro Spallanzani—IRCCS, 00149 Rome, Italy; isabella.abbate@inmi.it (I.A.); giulia.matusali@inmi.it (G.M.); elisabetta.lazzari@inmi.it (E.L.); fabrizio.maggi@inmi.it (F.M.); 3 Department of Experimental and Clinical Biomedical Sciences “Mario Serio”, University of Florence, 50121 Florence, Italy; michele.tanturli@unifi.it; 4 Clinical Department, National Institute for Infectious Diseases Lazzaro Spallanzani—IRCCS, 00149 Rome, Italy; marta.camici@inmi.it (M.C.); alessandra.vergori@inmi.it (A.V.); 5 Cellular Immunology and Pharmacology Laboratory, National Institute for Infectious Diseases Lazzaro Spallanzani—IRCCS, 00149 Rome, Italy; eleonora.cimini@inmi.it

**Keywords:** SARS-CoV-2, small RNA, microRNA, PASC, extracellular vesicles

## Abstract

The mechanisms underlying post-acute sequelae of SARS-CoV-2 infection (PASC) are a topic of debate. This study examined the presence of SARS-CoV-2 microRNA (miRNA)-like small RNAs in extracellular fluids and their potential link to PASC by using a quantitative stem-loop RT-PCR MiRNA assay. Initially, it was demonstrated that three previously identified SARS-CoV-2 miRNA-like small RNAs, specifically svRNA 1 and 2 and miR-07a, were significantly expressed in infected cells in vitro and released into the supernatant following infection by different SARS-CoV-2 variants. Then, the expression of three SARS-CoV-2 small RNAs was studied in both nasopharyngeal swabs (NPS) and sera from 24 patients at their initial COVID-19 diagnosis (T0) and in sera collected 91 to 193 days post-diagnosis (T1). Notably, 11 out of 24 patients (46%) reported PASC consequences. All NPS samples showed SARS-CoV-2 small RNA expression with an altered cytokine network during acute infection, but it did not correlate with PASC outcomes. Serum samples had similar small RNA statuses, though PASC patients, notably at T1, but not at T0, displayed reduced overall positivity compared to those without PASC. The host target expression of SARS-CoV-2 small RNAs was not significantly different between groups. This suggests a need for further research into SARS-CoV-2 small RNA and its role in viral behavior and PASC consequences.

## 1. Introduction

Since the advent of the severe acute respiratory syndrome coronavirus 2 (SARS-CoV-2) pandemic, increasing post-sequelae associated with the virus infection are a main human health concern [1]. It is well-documented that numerous instances of post-acute sequelae SARS-CoV-2 infection (PASC), commonly referred to as long coronavirus disease 19 (COVID-19), exist, though the underlying mechanisms remain unclear [2,3]. PASC is a multisystem condition affecting millions of individuals around the world, with an incidence of 10% of SARS-CoV-2 confirmed infected people worldwide, with the highest percentage in mild acute illness non-hospitalized patients [4]. There are likely numerous potential direct or indirect determinants of PASC, including persistent reservoirs of SARS-CoV-2, severe inflammation, immune dysregulation, impacts on the microbiota and its virome, microvascular blood clotting with endothelial dysfunction, and dysfunctional signaling in the brainstem [4,5].

So far, numerous studies have been carried out to gain a deeper insight into the molecular changes associated with SARS-CoV-2 infection. Among the recent molecular investigations, the more intriguing issue has been reported by the transcriptomic studies of SARS-CoV-2 infection. In this regard, interesting attention has been given to the expression and role of host and viral microRNAs (miRNA) [6,7]. It is well documented that many viruses can encode small non-coding RNAs including miRNA [6,8,9]. RNA viruses, unlike DNA viruses, can produce their microRNAs through a noncanonical biogenesis pathway [10]. Thus, RNA viruses replicating in cell cytoplasmic context hijack multiple strategies to bypass the requirement for nuclear Drosha and produce miRNA-like small RNA [10]. The interest in miRNA, non-coding RNAs 20 to 22 nucleotides in length, is related to their ability to silence gene expression. This can be performed by a transcript-specific target to mediate inhibitory activity, playing a key role in several cellular processes such as the replication, transcription, and translation of viral genomes as well as influencing immune evasion and inflammatory response [11]. Several RNAseq sequencing analyses of nasopharyngeal swabs (NPS) obtained from SARS-CoV-2-infected patients have reported the presence of putative viral-encoded small miRNA-like sequences [12,13,14,15,16]. Comparable findings, validated through real-time quantitative RT-PCR, have also been achieved in vitro by infecting various cellular substrates with SARS-CoV-2 [16,17,18,19,20,21,22,23]. Although the length of the sequence and distribution throughout the viral genome could be presumptive of degradation fragments rather than Dicer-generated miRNA-like molecules, the presence of the characteristic harping structure points out their effective presence [11]. Thus, several miRNA-like small molecules encoded by different genomic regions and utilizing cellular machinery independent of Drosha proteins, which exhibit a strong hairpin structure, have been identified [11]. Importantly, bioinformatic and biological studies have shown that virus-encoded small RNA primarily targets host immune response pathways, indicating that these small RNA may be involved in SARS-CoV2 pathogenesis [17,18,19].

Several investigations pointed towards possible viral persistence as a driver of PASC symptoms, reporting viral proteins and/or RNA presence in several tissues [24,25,26,27,28]. Of note, evidence suggests that human respiratory viruses can hijack extracellular vehicles (EVs) to deliver proteins, mRNAs, miRNAs, and whole viral particles during the viral life cycle in the host [29]. Thus, virus molecules delivered in such a way in biological fluids may increase the risk of inducing severe respiratory viruses-associated diseases in sites far from the respiratory tract and for a prolonged time [29]. This study investigated whether SARS-CoV-2 can produce small miRNA-like RNAs and deliver these in extracellular biological fluids. Initially, small miRNA-like RNA expressions were analyzed both in cells and extracellular fluids during infections with various SARS-CoV-2 variants. Specifically, three SARS-CoV-2 miRNA-like small RNAs, previously identified as originating from the SARS-CoV-2 intergenic *N* and *open reading frame* (*ORF) 10* genomic regions (svRNA 1 and svRNA 2 small RNA; [15]), along with the miR-07a miRNA derived from the SARS-CoV-2 ORF7a genomic region were examined [20]. Subsequently, circulating SARS-CoV-2 miRNA-like small RNAs were analyzed in NPS and sera at the time of the initial COVID-19 diagnosis in 24 patients and sera at different intervals (ranging from 91 to 193 days) for the same group of patients. Notably, 11 out of 24 (46%) participants were diagnosed with clinical parameters suggestive of PASC outcomes. An examination was conducted on some mRNA previously identified as possible target pathways of the specific SARS-CoV-2 small RNA to assess any correlation with the expression of the virus’s small RNA [15,20]. Subsequently, all findings were analyzed to explore potential significant differences between patients who showed PASC symptoms and those who did not.

## 2. Materials and Methods

Cells and viruses. The Vero E6 cell line (CRL-1586, American Type Culture Collection ATCC, Rockville, MD, USA) was grown in Dulbecco’s Modified Eagle’s Medium (DMEM) with 10% fetal bovine serum (FBS). The SARS-CoV-2 clinical isolates used were SCV2/Fi/3/22 (Wuhan-like B.1), SCV2/Fi/1/21 (Alpha-like B.1.1.7), SCV2/Fi/2/21 (Delta-like B.1.617.2), and SCV2/Fi/1/22 (Omicron-like BA.1).

Infection experiments. Vero E6 cells were seeded at a density of 7.5 × 10^5^ cells per well in a 24-well plate containing 2 mL of DMEM supplemented with 10% fetal bovine serum (FBS). After 24 h, achieving approximately 70% confluence, the cells were infected with SARS-CoV-2 at a multiplicity of infection (MOI) of 0.1 at 37 °C for 1 h. The virus inoculum was then removed and replaced with DMEM containing 10% FBS, followed by incubation at 37 °C. Samples of cells and supernatants were collected at 0, 3, 6, 24, and 48 h post-infection.

Real-time PCR analysis of RNA genomic sequences for SARS-CoV-2. RNA was isolated and purified from 150 microliters of supernatant, infected cells, NPS, and serum samples using the RNAeasy Mini Kit (Qiagen, Milan, Italy). Each sample’s total RNA (100 nanograms) was amplified in a 25 μL reaction mixture via one-step real-time PCR, utilizing primers targeting the N region: forward primer SC2-For 5′ CTG CAG ATT TGG ATG ATT TCT CC 3′; reverse primer SC2-Rev 5′ CCT TGT GTG GTC TGC ATG AGT TTA G 3′; and probe SC2-Probe FAM-5′ ATT GCA ACA ATC CAT GAG CAG TGC TGA 3′-MGB. Reactions were performed using a Rotor-Gene Q real-time machine (Corbett Research, Mortlake, Australia) with PCR Master Mix (Life Technologies, Foster City, CA, USA). The thermal cycling conditions included an initial denaturation at 95 °C for 10 min, followed by 40 cycles comprising 20 s at 95 °C and 60 s at 60 °C, with fluorescence recorded at 75 °C. All reactions were conducted in duplicate, and cycle threshold (Ct) values were determined using Rotor-Gene Q software version 2.3.1, discarding samples with a Ct difference of more than 1.0 between duplicates.

SARS-CoV-2 miRNA-like small RNA assay. Total RNA was isolated from 2.0 × 10^6^ Vero E6 cells using the mirVana miRNA isolation Kit (Ambion, Austin, TX, USA) and from 250 μL of cell-free supernatant using the extraction of circulating and exosome RNA kit (Norgen, Thorold, ON, Canada). The small RNA expression was measured and quantified with the specific (miR-07a UUCUUGGCACUGAUAACAC, svRNA 1 ACTCATGCAGACCACACAAGGCAG, svRNA2 CAAAACATTCCCACCAACAGAGCC) quantitative stem-loop RT-PCR MiRNA assay according to the manufacturer’s protocol. Each reaction was performed in triplicate using 100 ng of extracted RNA, including negative controls (no template). After the reactions, the Ct values were determined using the mean Ct values determined from triplicate PCRs in the control group. According to the detection spectrum of non-specific products, Ct values > 38 were set as the cut-off points for validation. Expression values were calculated using the ΔΔCt method and U6 snRNA, the most used endogenous control gene in miRNA. The expression value of uninfected control (Ctr), and the Ct value of the non-specific product detected in the PCR reaction were used.

Quantification of SERINC5, IFN-β, and CCL20 mRNA. To quantify serine incorporator protein 5 (SERINC5), interferon beta (IFN-β), and chemokine ligand 20 (CCL20) mRNA levels, one-step RT-qPCRs were performed. One hundred nanograms of total RNA were reverse-transcribed and amplified by qPCR in a 25 μL total volume reaction containing specific primers (SERINC5 Fwd ATCGAGTTCTGACGCTCTGC SERINC5 Rev GCTCTTCAGTGTCCTCTCCAC, IFN-β Fwd CATGAGCTACAACTTGCTTGG, IFN-β Rev TCCTCCTTCTGGAACTGCTG, CCL20 Fwd GCTTTGATGTCAGTGCTGCTAC CCL20 Rev TTGGATTTGCGCACACAG, RPP30 Fwd CTATTAATGTGGCGATTGACCGA RPP30 Rev TGAGGGCACTGGAAATTGTAT). Power SYBR Green PCR Master Mix, MultiScribe Reverse Transcriptase, and RNase Inhibitor (all from Applied Biosystems, Foster City, CA, USA), according to the manufacturer’s instructions. Relative quantitation of mRNA levels was calculated using the ΔΔCt method and ribonuclease RPP30 mRNAs as an endogenous control.

Cytokine quantification. Plasma samples from patients and controls were obtained after centrifugation for 20 min at 2000 rpm and stored at −80 °C. The inflammatory cytokines: Interferon-gamma Inducible Protein 10 (CXCL-10/IP-10), Interferon-gamma (IFN-γ), Interleukin-1β (IL-1 β), Interleukin-2 (IL-2) Interleukin-6 (IL-6), Interleukin-8 (IL-8), and Tumor Necrosis Factor-alpha (TNF-α), were quantified using an automated enzyme-linked immunosorbent assay (ELLA microfluidic analyzer, Protein Simple, Bio-techne, Minneapolis, MN, USA). Data are presented pg/mL. The quantification lower limits were: CXCL-10/IP-10 at 0.60 pg/mL, IFN-γ at 0.17 pg/mL, IL-1 β at 0.4 pg/mL, IL-2 at 0.54 pg/mL, IL-6 at 0.28 pg/mL, IL-8 at 0.19 pg/mL, and TNF-α at 0.30 pg/mL.

Patients and Samples. Clinical samples from 24 COVID-19 patients at INMI L. Spallanzani were collected during the acute phase (T0, NPS, and serum) and after a median of 151 days from diagnosis (T1, serum only). Eleven experienced PASC, reporting symptoms like asthenia, exertional dyspnea, cough, joint pain, and sleep disorders. Samples from age- and gender-matched healthy donors were also analyzed as controls. All participants provided informed consent, and the studies received approval from the Regional Ethical Committee (CET Lazio area 4) on 18 November 2021 (approval number 469/2021 for MONET) and on 4 May 2023 (approval number 18/2023 for DEGAS-PACS), respectively. Several samples were also residuals of diagnostic evaluations (CET approval number 61/2023, 4 December 2023).

Statistical analyses. The relationships between categorical variables were determined using Fisher’s exact test and, where appropriate, the Chi-Square test. For continuous variables, a non-parametric method was employed, utilizing the Wilcoxon/Mann–Whitney test and Kruskal–Wallis with results presented as medians and interquartile ranges (IQR). The Holm method was used in the case of multiple comparisons. Spearman tests were used for correlation analysis and linear regression was used to draw the line plot. A *p* value < 0.05 was considered statistically significant. All statistical analyses were performed using R software version 4.4.0 with R Studio 2024.04.1 Build 748. Moreover, data were analyzed using a two-tailed Student’s *t*-test. All data represent three independent experiments, and values represent the mean ± standard deviation (SD), with *p* < 0.05 considered statistically significant.

## 3. Results

**Figure 1:**
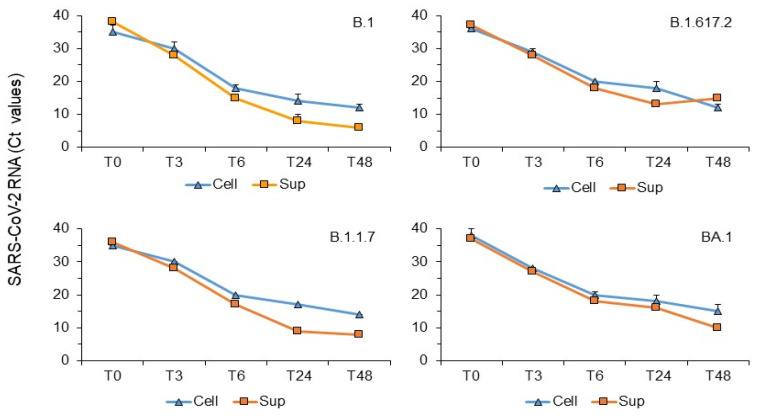
SARS-CoV-2 qPCR genomic RNA amplification performed on samples from Vero E6 cells infected with different viral variants. Cells were infected with SARS-CoV-2 variants (SCV2/Fi/3/22 (Wuhan-like B.1), SCV2/Fi/1/21 (Alpha B.1.1.7), SCV2/Fi/2/21 (Delta B.1.617.2), and SCV2/Fi/1/22 (Omicron BA.1)) at an MOI of 0.1. Samples were collected at T0, T3, T6, T24, and T48 h post-infection for RNA extraction. One hundred nanograms of total RNA were amplified using primers and probes targeting the SARS-CoV-2 *N* region. Mean + standard deviation values of the CT cycles were obtained from three independent experiments.

In vitro analysis of SARS-CoV-2 genomic replication and small RNA expression. Vero E6 cells were infected with SARS-CoV-2 at an MOI of 0.1 to study viral RNA replication and its small-RNA expression at intervals of 3, 6, 24, and 48 h post-infection. The kinetics of replication, monitoring the viral RNA *N* target genome in cells and their supernatant, showed no significant difference from 3 h post-infection, reaching high levels by 48 h (Figure 1).

**Figure 2:**
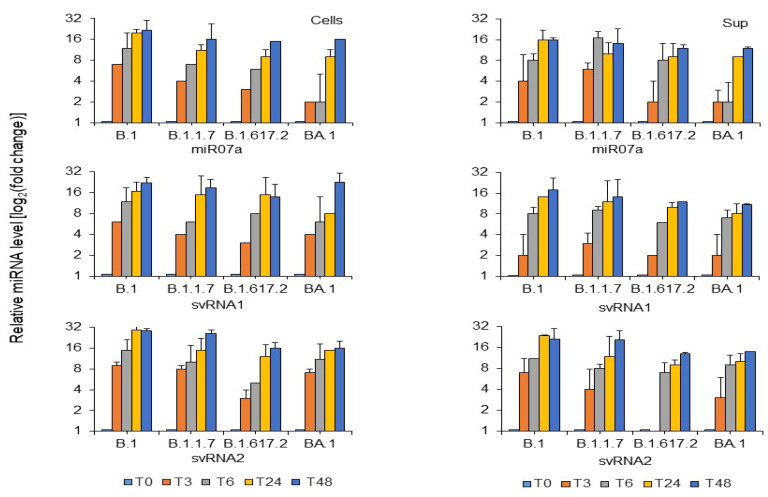
Relative expression of SARS-CoV-2 miRNA-like small RNA in Vero E6 cells measured at various times post-infection. Vero E6 cells were infected with SARS-CoV-2 variants (Wuhan-like B.1, Alpha-like B.1.1.7, Delta-like B.1.617.2, Omicron-like BA.1) at an MOI of 0.1. Samples from cells and supernatant (Sup) were collected at T0, T3, T6, T24, and T48 h after infection for RNA extraction. Using 100 ng of total RNA, RT-qPCR with specific primers was performed. Relative fold change was quantified via the ΔΔCt method with U6 snRNA as a control. Results are presented as mean + standard deviation from 3 independent experiments.

SARS-CoV-2 small RNAs svRNA 1, svRNA 2, and miR-07a were tracked in cellular contexts and extracellular supernatants. From 3 h post-infection, higher levels of svRNA 1, svRNA 2, and miR-07a were seen in the B.1 and B.1.1.7 variants compared to the B.1.617.2 and BA.1 variants (Figure 2).

Similar results were found in the extracellular supernatant, confirming the presence of SARS-CoV-2 small RNA in extracellular fluids (Figure 2).

In vivo analysis of SARS-CoV-2 genomic replication and small RNA expression. This part of the study was conducted to investigate the virus’s role in delivering viral miRNA within the host. NPS and serum samples from 24 COVID-19 patients were analyzed, revealing that only 2 (8%) showed SARS-CoV-2 RNA in serum at T0. Table 1 details the demographic, clinical, and virological characteristics.

**Figure 3:**
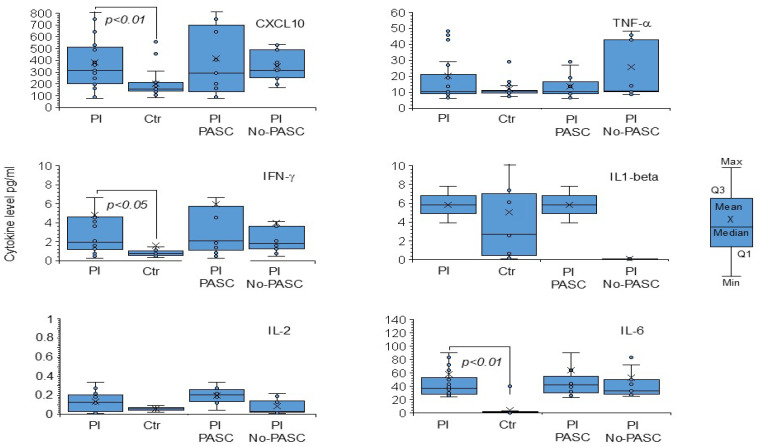
Cytokine levels in plasma samples collected at the initial SARS-CoV-2 positive diagnosis in study patients. Pl, all plasma samples, Pl PASC, plasma samples from patients with PASC, Pl No-PASC, plasma samples from patients without PASC. Ctr, plasma controls. Interferon-gamma Inducible Protein 10 (CXCL-10/IP-10), Interferon-gamma (IFN-γ), Interleukin-1β (IL-1 β), Interleukin-2 (IL-2) Interleukin-6 (IL-6), and Tumor Necrosis Factor-alpha (TNF-α). The box and whisker plots display the complete data set, indicating the minimum (min) and maximum (max) values, the first quartile (Q1), the third quartile (Q3), the median, and the mean for all the cytokines examined. Values reported are mean obtained in 3 independent experiments. Differences from control (n.17) values were found to be statistically significant at *p* < 0.01 Students *t*-test.

Of note, 11 out of 24 patients (46%) reported PASC consequences without significant clinical symptom differences. Elevated cytokine levels were observed in patient plasma samples at T0 compared to healthy controls (Figure 3), but no significant differences were found in age, gender, treatment, viral load in NPS, SARS-CoV-2 lineage, or median time from diagnosis at T1 between PASC and non-PASC patients (Table 1 and Table S1).

To verify the expression of SARS-CoV-2 small RNA in vivo, SARS-CoV-2 svRNA 1 and 2 and miR-07a were examined in NPS at the initial time (T0) of a positive viral diagnosis and compared with the viral miRNA expression in similar samples from control subjects obtained before the pandemic’s onset. Collectively, almost all 24 NPS samples were positive for the three small RNAs, with 23 out of 24 (95.8%) samples positive for SARS-CoV-2 miR-07 and 22 out of 24 (91.6%) samples positive for both svRNA 1 and 2. No significant differences were observed in the detection of each small RNA or overall positivity, considering at least one small RNA detected in NPS, between patients exhibiting PASC and those who did not (Table 2).

**Figure 4:**
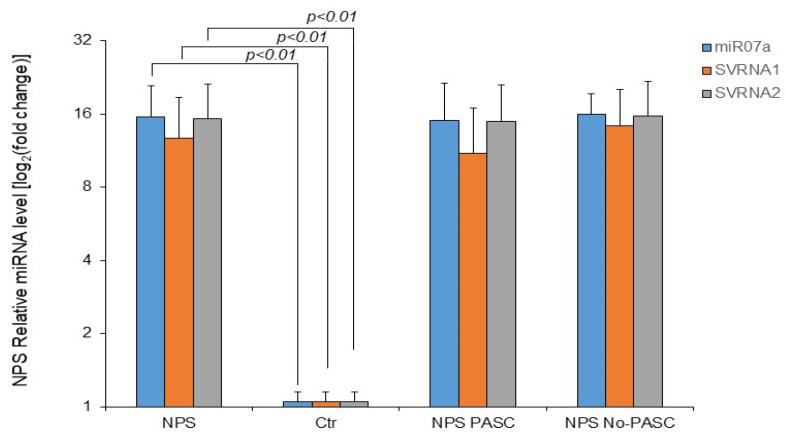
The relative expression of SARS-CoV-2 miRNA-like small RNA in NPS samples at various times following a positive diagnosis of SARS-CoV-2 analyzed in study patients. Total RNA (100 nanograms) underwent amplification using stem-loop small RNA-specific RT-qPCR. The ΔΔCt method determined the relative fold change quantification, utilizing U6 snRNA as an internal control. NPS, all nasopharyngeal swab samples, NPS PASC, nasopharyngeal swab samples from patients with PASC, NPS No-PASC, nasopharyngeal swab samples from patients without PASC, Ctr, NPS controls. The reported values are the mean + standard deviation from three independent experiments. Statistically significant differences from control values (n = 20) were identified at *p* < 0.01 (Student’s *t*-test).

As shown in Figure 4, the mean small RNA expression in NPS samples of study patients was significantly higher for all three small RNAs compared to control samples. Interestingly, no difference in expression levels was detected between patients with or without PASC. Given that SARS-CoV-2 small RNA targets are related mainly to SERINC5 (for svRNA1 and 2) and the Interferon pathway (for all three small RNAs), the expression of SERINC5, IFN-β, and CCL20 mRNA in NPS of study patients was further investigated.

**Figure 5:**
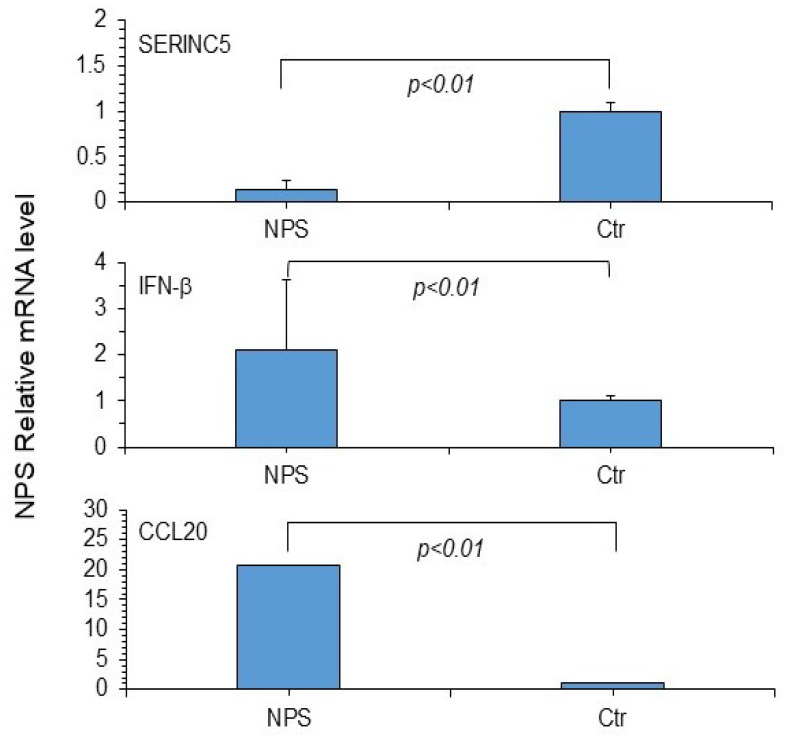
Expression levels of SERINC5, IFN-β, and CCL20 mRNAs in NPS samples from study patients. For relative quantification of these markers, the ΔΔCt method was applied, using RPP30 mRNA as an endogenous control [15]. Ctr, NPS controls. Serine incorporator protein 5 (SERINC5), interferon beta (IFN-β), and chemokine ligand 20 (CCL20). The results are presented as mean + standard deviation from at least three experiments. Differences from control values (n = 20) were statistically significant at *p* < 0.01 (Student’s *t*-test).

Figure 5 illustrates that SERINC5 expression in NPS samples from study patients was significantly lower compared to control samples. Furthermore, the study patients’ samples showed elevated levels of IFN-β and CCL20 expression relative to control samples. When various parameters were analyzed in NPS at the time of initial SARS-CoV-2 positive diagnosis, a significant correlation was observed exclusively between SARS-CoV-2 small RNA expression and viral yield (see Table S2).

**Figure 6:**
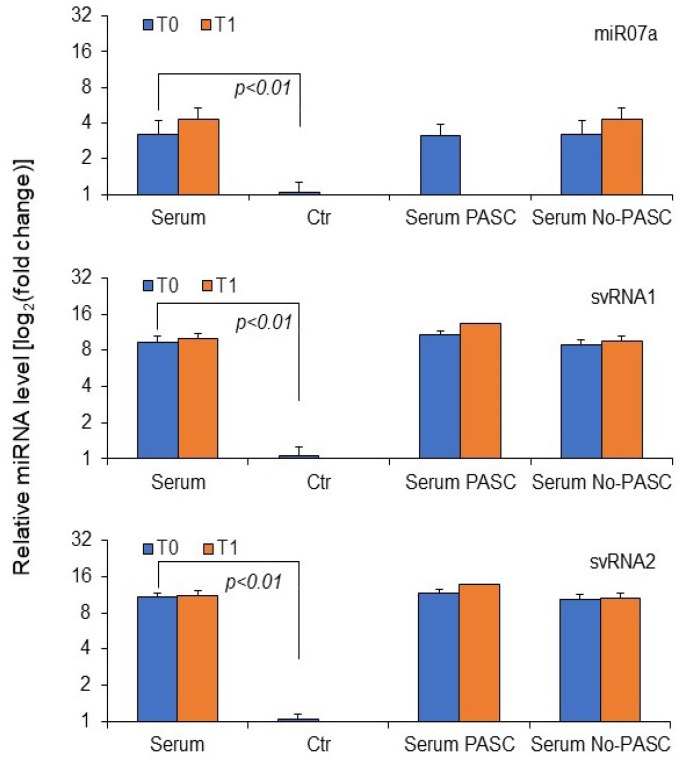
The relative expression of SARS-CoV-2 microRNA-like small RNA in serum samples assessed in study patients. A total of 100 nanograms of RNA was amplified using steam-loop small RNA-specific RT-qPCR. The ΔΔCt method quantified the relative fold change, employing U6 snRNA as an internal control [15]. Serum, all serum samples, Serum PASC, serum samples from patients with PASC, Serum No-PASC, serum samples from patients without PASC. Ctr, serum controls. T0, time at the first SARS-CoV-2 positive diagnosis. T1, time from diagnosis (92–193 days). Reported values represent the mean + standard deviation from three independent experiments. Differences from control values (n = 20) were statistically significant with a *p*-value of less than 0.01, as determined by Student’s *t*-test.

It is well known that miRNAs linked to extracellular vesicles or proteins can be found in extracellular biological fluids. Although serum SARS-CoV-2 RNA genomic positivity was noted in only two patients at diagnosis, the presence of SARS-CoV-2 miRNA-like small RNA in serum samples at initial and subsequent positive diagnoses for PASC was investigated. Initially, 10 out of 24 (41.7%) serum samples showed at least one viral small RNA, with 2 out of 24 (8.3%) being positive for miR-07a, and 10 out of 24 (41.7%) showing both svRNA 1 and 2. At the second detection, 8 out of 24 (33%) were positive for at least one viral small RNA, with 2 out of 24 (8.3%) positive for miR-07a, and 6 out of 24 (25%) positive for both svRNA 1 and 2. Only one serum sample tested positive for SARS-CoV-2 RNA genomics. No significant differences were found in viral small RNA serum positivity between T0 and T1, but total sera positivity for at least one viral small RNA at long-term (T1) diagnosis was significantly lower in PASC patients than non-PASC ones (*p* = 0.033 vs. *p* = 0.240, for total small RNA at time T1 and T0, respectively, Fisher test, Table 2). Figure 6 indicates higher expression levels of all three SARS-CoV-2 small RNAs compared to control sera, with no differences between PASC and non-PASC patients. 

**Figure 7:**
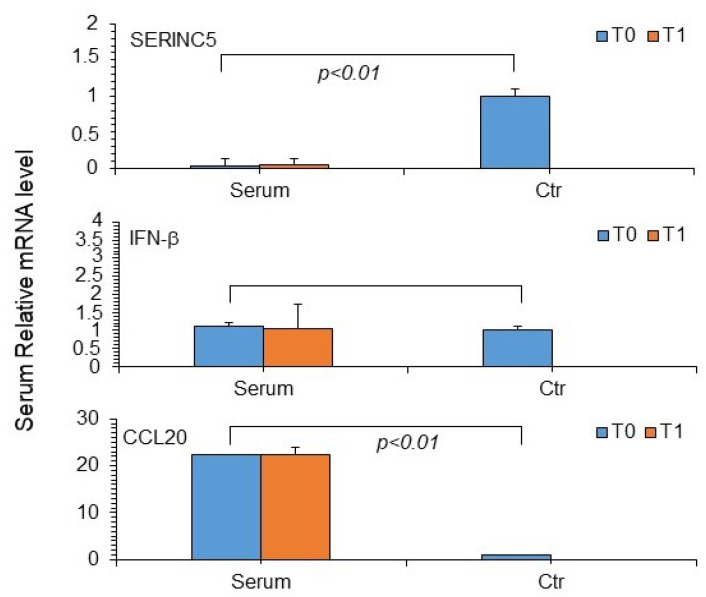
Expression levels of SERINC5, IFN-β, and CCL20 mRNAs in the serum samples of the study patients. For relative quantification of these markers, the ΔΔCt method was applied, using RPP30 mRNA as an endogenous control [15]. Ctr, serum controls. Serine incorporator protein 5 (SERINC5), interferon beta (IFN-β), and chemokine ligand 20 (CCL20). T0, time at the first SARS-CoV-2 positive diagnosis. T1, time from diagnosis (92–193 days). Reported values represent the mean + standard deviation from a minimum of three independent experiments. Statistical analysis revealed significant differences from the control group (n = 20) at *p* < 0.01 using Student’s *t*-test.

SERINC5 mRNA showed reduced expression, whereas IFN-β and CCL20 mRNA levels were elevated in patient serum samples at both T0 and T1 compared to controls (Figure 7). However, no significant correlation was observed among different parameters including cytokine expression levels observed in patients with or without PASC (Table S3).

## 4. Discussion

In this study, the expression of three SARS-CoV-2 miRNA-like small RNAs was examined during cell infection and in paired NPS and serum samples from 24 patients infected with SARS-CoV-2. The results indicated that the three selected SARS-CoV-2 small RNAs, previously identified within the cellular substrate, were expressed in cells and delivered in the extracellular fluid supernatant derived from infected cells. Moreover, the three chosen SARS-CoV-2 small RNAs were consistently expressed across different SARS-CoV-2 variants without statistically significant differences. These findings confirm the ability of SARS-CoV-2 to express small RNAs during infection and highlight its potential to be transported in biological fluids, either associated with extracellular vesicles or within a protein context, like other viral miRNAs. The lack of variant-associated differences in their expression suggests these viral molecules might be a conserved feature of SARS-CoV-2, needing further investigation for their role in host-viral interactions and post-infection effects. Thus, we examined the presence of three small RNAs expressed by SARS-CoV-2 in NPS and serum samples from 11 patients with PASC symptoms and 13 without, both at diagnosis and over a prolonged period (median range 92 to 193 days). The data confirmed the expression of small RNAs in nearly all nasal swabs along with an altered cytokine network during acute infection, unrelated to PASC induction. In serum samples, 42% of SARS-CoV-2 small RNA positivity at acute infection reduced to 33% over time, without viral RNA viremia. This suggests the viral small RNAs were likely delivered from the respiratory tract into extracellular fluid. Although individual viral small RNA status did not differ between PASC and non-PASC patients, overall positivity for at least one small RNA was lower in PASC patients at prolonged follow-up but not at initial diagnosis. Additionally, viral small RNA expression levels did not significantly differ between PASC and non-PASC patients. The limited samples prevented the analysis of any correlation between SARS-CoV-2 small RNA expression and viral lineage in PASC and non-PASC.

Currently, the investigation of biological markers to understand the events leading to PASC is a key concern for human health. Notably, the persistence of SARS-CoV-2 in various tissue compartments is commonly explored to clarify this viral impact [1,2,3]. Extensive studies have indicated prolonged SARS-CoV-2 positivity in the upper respiratory tract (with an average duration of 17 days and a maximum of 83 days; [30]). In addition, viral RNA has been detected in several samples (feces, plasma, and urine) of patients up to 7 months post-infection, and its potential integration has also been considered [31]. Moreover, persistent circulating SARS-CoV-2 spike protein has been identified in the plasma of PASC patients up to 12 months following infection [26,27]. Several studies have reported the presence of SARS-CoV-2 encoded small RNA, though their role remains not fully understood [12,13,14,15,16]. The results obtained in our study indicate that the three specific viral small RNA expression profiles, and their effects on the host immune response factor, are not involved in PASC sequelae. Additionally, no significant differences were found in age, gender, treatment, viral load, or time from diagnosis between PASC and non-PASC patients. However, a reduced positivity in sera for the three viral small RNAs, possibly due to altered miRNA-like RNA processing, may be a consequence of PASC. This indicates that changes in miRNA processing may occur in PASC patients.

Like other viral miRNAs, SARS-CoV-2 small RNAs likely interact with viral elements to impact replication and gene expression, as well as with host components to influence inflammation pathways [9]. Although the effect on inflammation is well documented, the exact targets and mechanisms of viral inhibition are still not fully understood [14,15,16,17,18,19,20,21,22]. So far, the identified SARS-CoV-2 small RNAs are mostly produced from “hotspots” near the genome’s ends [14,15,22]. Notably, many of these small RNAs originate from *ORF8*, *ORF7*, and the *N* gene. Furthermore, elevated levels of subgenomic RNA encoding this gene have been observed in various tissues for extended periods post-infection [32]. The involvement of this gene, from which the small RNA is derived, in multiple mechanisms of SARS-CoV-2 mediated diseases has also been documented [33,34]. Further molecular investigations are needed to understand the role of SARS-CoV-2 small RNA in PASC patients. These investigations could help clarify whether SARS-CoV-2 small RNA plays a part in a viral mechanism that targets complementary encoding or regulatory regions, potentially affecting viral replication or gene expression. Consequently, a decrease in the expression of these viral small RNAs could be linked to molecular reactivation of the virus, which may contribute to PASC outcomes.

Due to the difficulties in enrolling SARS-CoV-2 positive patients with clear symptoms representing PASC status, the small sample size used is a primary limitation of this study. Additionally, the lack of investigation into other SARS-CoV-2 microRNAs is also a limitation. Although studies have reported additional SARS-CoV-2 small RNA [12,13,14,15,16], their expression has not yet been clearly demonstrated. However, it is crucial to thoroughly investigate the role of small RNA in SARS-CoV-2 to comprehend its impact on viral properties and to uncover additional factors that contribute to the onset of PASC complications.

## Figures and Tables

**Table 1:** Demographical, clinical, and viral features of COVID-19 patients.

	All (n = 24)	PASC (n = 11)	No PASC (n = 13)	*p* Value *
Age: median (IQR)	69 (54–73)	69 (58–72)	69 (54–76)	0.7867
Gender: M/F	10/14	4/7	6/7	0.6968
Treatment n (%):				
Evusheld	5 (21)	2 (18)	3 (23)	>0.9999
Sotrovimab	11(46)	5 (45)	6 (46)	>0.9999
Paxlovid	8 (33)	4 (36)	4 (31)	>0.9999
SARS-CoV2 RNA in NPS				
Median Ct values (IQR)	14.9 (13.1–18.3)	13.5 (12.8–16.9)	15.7 (13.4–20.2)	0.1799
SARS-CoV2-lineage n. (%)				
BA.2	13(54.16)	3 (27)	3 (23)	>0.9999
BA.4	1(4.16)	1 (9)	0	0.4583
BA.5	7(29.16)	1 (9)	6 (46)	0.0778
BF.5	1(4.16)	0	1 (8)	>0.9999
unknown	2(8.33)	2 (18)	0	0.1993
T1 median days from diagnosis (IQR)	151 (92–193)	181 (107–196)	111 (92–183)	0.1543

* PASC vs. no PASC. NPS, nasopharyngeal swab.

**Table 2:** SARS-CoV-2 small RNA positivity status.

		All (n = 24)	PASC (n = 11)	No PASC (n = 13)	*p* Value *
NPS miR07a at T0	Negative (%)	1 (4.2)	0 (0)	1 (7.7)	0.999
	Positive (%)	23 (95.8)	11 (100)	12 (92.3)	
NPS svRNA 1 at T0	Negative (%)	2 (8.4)	0 (0)	2 (15.4)	0.565
	Positive (%)	22 (91.6)	11 (100)	11 (84.6)	
NPS svRNA 2 at T0	Negative (%)	2 (8.4)	0 (0)	2 (15.4)	0.565
	Positive (%)	22 (91.6)	11 (100)	11 (84.6)	
Total NPS viral small RNA at T0					
	Negative (%)	1 (4.2)	0 (0)	1 (7.7)	0.999
	Positive (%)	23 (95.8)	11 (100)	12 (92.3)	
Serum miR07a at T0	Negative (%)	22 (91.6)	10 (90.9)	12 (92.4)	0.999
	Positive (%)	2 (8.4)	1 (9.1)	1 (7.6)	
Serum svRNA 1 at T0	Negative (%)	14 (58.3)	8 (72.7)	6 (46.2)	0.369
	Positive (%)	10 (41.7)	3 (27.3)	7 (53.8)	
Serum svRNA 2 at T0	Negative (%)	14 (58.3)	8 (72.7)	6 (46.2)	0.369
	Positive (%)	10 (41.7)	3 (27.3)	7 (53.8)	
Total serum viral small RNA at T0					
	Negative (%)	14 (58.3)	8 (72.7)	6 (46.2)	0.240
	Positive (%)	10 (41.7)	3 (27.3)	7 (53.8)	
Serum miR07a at T1	Negative (%)	22 (91.6)	11 (100)	11 (84.6)	0.565
	Positive (%)	2 (8.4)	0 (0)	2 (15.4)	
Serum svRNA 1 at T1	Negative (%)	18 (75.0)	10 (90.9)	8 (61.5)	0.236
	Positive (%)	6 (25.0)	1 (9.1)	5 (38.5)	
Serum svRNA 2 at T1	Negative (%)	18 (75.0)	10 (90.9)	8 (61.5)	0.236
	Positive (%)	6 (25.0)	1 (9.1)	5 (38.5)	
Total serum viral small RNA at T1					
	Negative (%)	16 (66.7)	10 (90.9)	6 (46.2)	0.033
	Positive (%)	8 (33.3)	1 (9.1)	7 (53.8)	

* PASC vs. no PASC. NPS, nasopharyngeal swab. T0, time at the first SARS-CoV-2 positive diagnosis. T1, time from diagnosis (92–193 days).

## Data Availability

The original contributions presented in this study are included in the article/Supplementary Material. Further inquiries can be directed to the corresponding author.

## Supplementary Materials

The following supporting information can be downloaded at: https://www.mdpi.com/article/10.3390/microorganisms13010126/s1, Table S1. Statistical analysis of study patient’s viro-immunological data; Table S2. Statistical correlation analysis of study patient’s data in nasopharyngeal swab at the first time of SARS-CoV-2 positivity; Table S3. Statistical correlation analysis of study patient’s data in serum and plasma samples.

## Abbreviations

The following abbreviations are used in this manuscript:

PASC	post-acute sequelae of SARS-CoV-2 infection
MiRNA	microRNA
COVID-19	coronavirus disease 19
SARS-CoV-2	severe acute respiratory syndrome coronavirus 2
NPS	nasopharyngeal swabs
EV	extracellular vehicles
ORF	open reading frame
ATTC	American Type Culture Collection
DMEM	Dulbecco’s Modified Eagle’s Medium
FBS	fetal bovine serum
MOI	multiplicity of infection
SERINC5	serine incorporator protein 5
IFNβ	interferon beta
CCL20	chemokine ligand 20
CXCL-10/IP-10	Interferon-gamma Inducible Protein 10
IFN-γ	Interferon-gamma
IL-1 β	Inter-leukin-1β
IL-2	Interleukin-2
IL-6	Interleukin-6
IL-8	Interleukin-8
TNF-α	Tumor Necrosis Factor-alpha
IQR	interquartile ranges
SD	standard deviation
Ctr	control
