# Supplementary material for: Reduced Presence of SARS-CoV-2 microRNA-like Small RNA in the Serum of Patients with Post-Acute Sequelae SARS-CoV-2 Infection
Source: Microorganisms. 2025 Jan 9;13(1):126. doi: 10.3390/microorganisms13010126 (PMC11767842; doi:10.3390/microorganisms13010126)
Supplement: Supplementary file 1 [file microorganisms-13-00126-s001.zip › microorganisms-3412034-supplementary.pdf]

Table S1. Statistical analysis of study patient's viro-immunological data[a]

	Overall (24)	PASC (11)	No PASC (13)	*p*
NPS ct RNA (median [IQR])	14.85 [13.09, 17.52]	13.52 [12.88, 16.83]	15.75 [13.56, 20.09]	0.171
Plasma_IFN-γ at T0 (median [IQR])"	1.97 [1.16, 4.58]	1.77 [1.12, 3.84]	1.97 [1.44, 7.86]	0.500
Plasma_CXCL10 at T0 (median [IQR])"	317.50 [200.75, 510.00]	283.50 [193.00, 435.00]	337.50 [238.25, 512.00]	0.668
Plasma_IL-2 at T0 (median [IQR])"	0.03 [0.00, 0.16]	0.08 [0.02, 0.16]	0.01 [0.00, 0.14]	0.345
Plasma_TNF-α at T0 (median [IQR])"	10.45 [9.16, 21.10]	10.35 [9.16, 32.40]	10.60 [9.14, 15.30]	0.668
Plasma_IL-1β at T0 (median [IQR])"	0.00 [0.00, 0.00]	0.00 [0.00, 0.00]	0.00 [0.00, 0.00]	0.655
Plasma_IL-6 at T0 (median [IQR])"	37.00 [28.00, 53.50]	35.50 [29.50, 48.25]	38.50 [28.00, 55.50]	0.736
NPS miR07 at T0 (median [IQR])	16.12 [13.77, 19.32]	15.68 [13.34, 18.34]	17.31 [13.96, 19.57]	0.460
NPS SVRNA1 at T0 (median [IQR])	14.25 [10.49, 16.95]	11.05 [8.05, 15.78]	14.96 [13.04, 17.94]	0.175
NPS SVRNA2 at T0 (median [IQR])	17.78 [13.69, 19.25]	17.78 [12.53, 19.44]	17.89 [14.23, 19.15]	0.926
Serum_miR07 at T0 (median [IQR])	0.00 [0.00, 0.00]	0.00 [0.00, 0.00]	0.00 [0.00, 0.00]	0.952
Serum_SVRNA1 at T0 (median [IQR])	0.00 [0.00, 8.64]	0.00 [0.00, 3.98]	2.50 [0.00, 11.21]	0.286
Serum_SVRNA2 at T0 (median [IQR])	0.00 [0.00, 9.15]	0.00 [0.00, 4.21]	3.54 [0.00, 11.35]	0.286
Serum_miR07 at T1 (median [IQR])	0.00 [0.00, 0.00]	0.00 [0.00, 0.00]	0.00 [0.00, 0.00]	0.184
Serum_SVRNA1 at T1 (median [IQR])	0.00 [0.00, 1.82]	0.00 [0.00, 0.00]	0.00 [0.00, 7.59]	0.159
Serum_SVRNA2 at T1 (median [IQR])	0.00 [0.00, 1.80]	0.00 [0.00, 0.00]	0.00 [0.00, 7.19]	0.137
NPS miR07 at T0 (mean [SD])	15.52 (5.31)	14.98 (4.63)	16.01 (6.03)	0.655
NPS SVRNA1 at T0 (mean [SD])	12.74 (5.96)	11.03 (6.14)	14.30 (5.60)	0.197
NPS SVRNA2 at T0 (mean [SD])	15.31 (5.94)	14.92 (6.52)	15.67 (5.63)	0.771
Serum_miR07 at T0 (mean [SD])	0.26 (0.90)	0.28 (0.94)	0.25 (0.90)	0.930
Serum_SVRNA1 at T0 (mean [SD])	3.93 (5.24)	2.92 (5.15)	4.78 (5.36)	0.397
Serum_SVRNA2 at T0 (mean [SD])	4.48 (6.14)	3.17 (5.58)	5.59 (6.58)	0.347
Serum_miR07 at T1 (mean [SD])	0.36 (1.22)	0.00 (0.00)	0.66 (1.62)	0.190
Serum_SVRNA1 at T1 (mean [SD])	2.52 (4.58)	1.21 (4.01)	3.63 (4.89)	0.204
Serum_SVRNA2 at T1 (mean [SD]])	2.77 (5.18)	1.24 (4.13)	4.07 (5.77)	0.190

a T0, time at the first SARS-CoV-2 positive diagnosis. T1, time between 3 to 9 months after the first time. NPS, nasopharyngeal swab. CXCL-10/IP-10, Interferon-gamma Inducible Protein 10; IFN-γ, Interferon-gamma; IL-1 β, Interleukin-1β, IL-2, Interleukin-2; IL-6, Interleukin-6; TNF-α, Tumor Necrosis Factor-alpha.

Table S2. Statistical correlation analysis of study patient's data in nasopharyngeal swab at the first time of SARS-CoV-2 positivity

Parameter1	Parameter2	rho	CI	CI_low	CI_high	S	p[a]	n_Obs
NPS miR07	NPS RNA_CT	-0.45	0.95	-0.74	-0.01	2234.00	0.040	21
NPS miR07	NPS SERINC5	-0.34	0.95	-0.67	0.10	2707.36	0.115	23
NPS miR07	NPS IFN-β	-0.04	0.95	-0.45	0.39	2098.00	0.868	23
NPS miR07	NPS CCL20	0.00	0.95	-0.42	0.42	2028.00	0.993	23
NPS SVRNA1	NPS RNA_CT	-0.12	0.95	-0.53	0.34	1720.23	0.613	21
NPS SVRNA1	NPS SERINC5	-0.29	0.95	-0.64	0.15	2615.79	0.176	23
NPS SVRNA1	NPS IFN-β	-0.43	0.95	-0.72	-0.01	2892.86	0.041	23
NPS SVRNA1	NPS CCL20	0.06	0.95	-0.37	0.47	1901.88	0.784	23
NPS SVRNA2	NPS RNA_CT	-0.43896	0.95	-0.74	0.00	2216.00	0.05	21
NPS SVRNA2	NPS SERINC5	-0.30	0.95	-0.64	0.14	2632.96	0.163	23
NPS SVRNA2	NPS IFN-β	-0.29	0.95	-0.64	0.15	2618.15	0.174	23
NPS SVRNA2	NPS CCL20	-0.01	0.95	-0.43	0.42	2038.00	0.975	23

NPS, nasopharyngeal swab, SERINC5, Serine incorporator protein 5, IFN-β, interferon beta, CCL20, chemokine ligand 20.

Table S3. Statistical correlation analysis of study patient's data in serum and plasma samples[a]

Parameter1	Parameter2	rho	CI	CI_low	CI_high	S	*p*	n_Obs
serum at t0 miR07	serum at t0 SERINC5	-0.11355	0.95	-0.5038	0.315191	2561.171	0.597284	24
serum at t0 miR07	serum at t0 IFN-β	0.097911	0.95	-0.32937	0.491904	2074.804	0.648998	24
serum at t0 miR07	serum at t0 CCL20	-0.36815	0.95	-0.67866	0.054005	3146.753	0.076716	24
serum at t0 miR07	serum at t1 SERINC5	-0.13402	0.95	-0.51915	0.296352	2608.237	0.532417	24
serum at t0 miR07	serum at t1 IFN-β	0.19228	0.95	-0.24082	0.561517	1857.757	0.368052	24
serum at t0 miR07	serum at t1 CCL20	0.164092	0.95	-0.26804	0.541257	1922.589	0.443567	24
serum at t0 miR07	plasma at t0 CXCL10	0.322744	0.95	-0.10524	0.649857	1557.689	0.123997	24
serum at t0 miR07	plasma at t0 IFN-γ	0.058034	0.95	-0.36465	0.460891	2166.522	0.787655	24
serum at t0 miR07	plasma at t0 IL-2	0.158304	0.95	-0.27355	0.537043	1935.9	0.460024	24
serum at t0 miR07	plasma at t0 TNF-α	-0.19319	0.95	-0.56216	0.239928	2744.329	0.365754	24
serum at t0 miR07	plasma at t0 IL-1β	-0.09074	0.95	-0.4864	0.335804	2508.696	0.673272	24
serum at t0 miR07	plasma at t0 IL-6	0.135258	0.95	-0.2952	0.520069	1988.907	0.528592	24
serum at t0 SVRNA1	serum at t0 SERINC5	-0.30402	0.95	-0.6377	0.125735	2999.239	0.148652	24
serum at t0 SVRNA1	serum at t0 IFN-β	0.017476	0.95	-0.39934	0.428307	2259.805	0.935402	24
serum at t0 SVRNA1	serum at t0 CCL20	-0.22991	0.95	-0.58789	0.203376	2828.786	0.279811	24
serum at t0 SVRNA1	serum at t1 SERINC5	-0.14689	0.95	-0.52867	0.284331	2637.836	0.493405	24
serum at t0 SVRNA1	serum at t1 IFN-β	-0.31616	0.95	-0.6456	0.11249	3027.169	0.1323	24
serum at t0 SVRNA1	serum at t1 ISG20	-0.25728	0.95	-0.60661	0.175315	2891.755	0.22486	24
serum at t0 SVRNA1	serum at t1 CCL20	-0.04466	0.95	-0.45027	0.376223	2402.72	0.835842	24
serum at t0 SVRNA1	plasma at t0 CXCL10	0.276702	0.95	-0.15498	0.619659	1663.585	0.19056	24
serum at t0 SVRNA1	plasma at t0 IFN-γ	0.353965	0.95	-0.07026	0.669763	1485.881	0.089708	24
serum at t0 SVRNA1	plasma at t0 IL-2	0.347043	0.95	-0.07811	0.665388	1501.802	0.096614	24
serum at t0 SVRNA1	plasma at t0 TNF-α	0.022097	0.95	-0.39545	0.432074	2249.176	0.91837	24
serum at t0 SVRNA1	plasma at t0 IL-1β	-0.24293	0.95	-0.59684	0.190114	2858.744	0.252696	24
serum at t0 SVRNA1	plasma at t0 IL-6	0.026248	0.95	-0.39194	0.435446	2239.629	0.903101	24
serum at t0 SVRNA2	serum at t0 SERINC5	-0.30402	0.95	-0.6377	0.125735	2999.239	0.148652	24
serum at t0 SVRNA2	serum at t0 IFN-β	0.033981	0.95	-0.38537	0.441697	2221.844	0.874748	24
serum at t0 SVRNA2	serum at t0 CCL20	-0.26292	0.95	-0.61042	0.169446	2904.726	0.214502	24
serum at t0 SVRNA2	serum at t1 SERINC5	-0.14689	0.95	-0.52867	0.284331	2637.836	0.493405	24
serum at t0 SVRNA2	serum at t1 IFN-β	-0.29285	0.95	-0.63037	0.137781	2973.553	0.164911	24

serum at t0 SVRNA2	serum at t1 CCL20	-0.03204	0.95	-0.44013	0.387022	2373.69	0.881855	24
serum at t0 SVRNA2	plasma at t0 CXCL10	0.301945	0.95	-0.12798	0.636342	1605.526	0.151578	24
serum at t0 SVRNA2	plasma at t0 IFN-γ	0.374358	0.95	-0.04683	0.682524	1438.977	0.071506	24
serum at t0 SVRNA2	plasma at t0 IL-2	0.320117	0.95	-0.10814	0.648161	1563.731	0.127264	24
serum at t0 SVRNA2	plasma at t0 TNF-α	-0.00219	0.95	-0.41574	0.412116	2305.027	0.991914	24
serum at t0 SVRNA2	plasma at t0 IL-1β	-0.24293	0.95	-0.59684	0.190114	2858.744	0.252696	24
serum at t0 SVRNA2	plasma at t0 IL-6	0.025276	0.95	-0.39276	0.434657	2241.865	0.906674	24
serum at t0 SERINC5	serum at t0 IFN-β	-0.18153	0.95	-0.55384	0.251283	2717.511	0.395933	24
serum at t0 SERINC5	serum at t0 CCL20	-0.05598	0.95	-0.45927	0.366437	2428.761	0.795001	24
serum at t0 SERINC5	serum at t1 miR07	0.285459	0.95	-0.14568	0.625481	1643.444	0.176331	24
serum at t0 SERINC5	serum at t1 SVRNA1	-0.21476	0.95	-0.57737	0.218599	2793.953	0.313564	24
serum at t0 SERINC5	serum at t1 SVRNA2	-0.21484	0.95	-0.57742	0.218519	2794.139	0.313378	24
serum at t0 SERINC5	serum at t1 SERINC5	0.210807	0.95	-0.22254	0.574597	1815.143	0.322771	24
serum at t0 SERINC5	serum at t1 IFN-β	-0.07567	0.95	-0.47473	0.349197	2474.039	0.725276	24
serum at t0 SERINC5	serum at t1 CCL20	0.113454	0.95	-0.31528	0.503727	2039.055	0.597605	24
serum at t0 SERINC5	plasma at t0 CXCL10	0.234472	0.95	-0.19875	0.591038	1760.714	0.270107	24
serum at t0 SERINC5	plasma at t0 IFN-γ	0.202749	0.95	-0.23053	0.568931	1833.678	0.342032	24
serum at t0 SERINC5	plasma at t0 IL-2	0.169364	0.95	-0.263	0.54508	1910.462	0.428851	24
serum at t0 SERINC5	plasma at t0 TNF-α	0.272409	0.95	-0.15951	0.61679	1673.46	0.197815	24
serum at t0 SERINC5	plasma at t0 IL-1β	-0.11355	0.95	-0.5038	0.315191	2561.171	0.597284	24
serum at t0 SERINC5	plasma at t0 IL-6	0.146926	0.95	-0.28429	0.528701	1962.071	0.493284	24
serum at t0 IFN-β	serum at t0 CCL20	-0.31137	0.95	-0.64249	0.117731	3016.156	0.138585	24
serum at t0 IFN-β	serum at t1 miR07	0.004533	0.95	-0.41017	0.417678	2289.574	0.983229	24
serum at t0 IFN-β	serum at t1 SVRNA1	-0.17375	0.95	-0.54825	0.258793	2699.621	0.416818	24
serum at t0 IFN-β	serum at t1 SVRNA2	-0.15209	0.95	-0.53249	0.279433	2649.8	0.478052	24
serum at t0 IFN-β	serum at t1 SERINC5	-0.1185	0.95	-0.50754	0.310666	2572.553	0.581295	24
serum at t0 IFN-β	serum at t1 IFN-β	0.286646	0.95	-0.14442	0.626268	1640.713	0.174461	24
serum at t0 IFN-β	serum at t1 CCL20	-0.31739	0.95	-0.6464	0.111139	3030	0.130718	24
serum at t0 IFN-β	plasma at t0 CXCL10	0.113043	0.95	-0.31566	0.503417	2040	0.59894	24
serum at t0 IFN-β	plasma at t0 IFN-γ	0.173081	0.95	-0.25943	0.547766	1901.913	0.418638	24
serum at t0 IFN-β	plasma at t0 IL-2	-0.18489	0.95	-0.55625	0.248022	2725.242	0.387093	24
serum at t0 IFN-β	plasma at t0 TNF-α	0.070465	0.95	-0.35378	0.470666	2137.93	0.743526	24
serum at t0 IFN-β	plasma at t0 IL-1β	-0.0281	0.95	-0.43695	0.390366	2364.639	0.896284	24
serum at t0 IFN-β	plasma at t0 IL-6	-0.08272	0.95	-0.4802	0.342955	2490.248	0.70079	24

serum at t0 CCL20	serum at t1 miR07	0.116068	0.95	-0.31289	0.505701	2033.044	0.589134	24
serum at t0 CCL20	serum at t1 SVRNA1	0.300124	0.95	-0.12995	0.635148	1609.715	0.154184	24
serum at t0 CCL20	serum at t1 SVRNA2	0.247624	0.95	-0.1853	0.600049	1730.465	0.243362	24
serum at t0 CCL20	serum at t1 SERINC5	0.247769	0.95	-0.18515	0.600147	1730.132	0.243078	24
serum at t0 CCL20	serum at t1 IFN-β	0.296715	0.95	-0.13363	0.632911	1617.555	0.159147	24
serum at t0 CCL20	serum at t1 CCL20	0.306588	0.95	-0.12294	0.639377	1594.847	0.145075	24
serum at t0 CCL20	plasma at t0 CXCL10	-0.18961	0.95	-0.55961	0.243428	2736.095	0.374875	24
serum at t0 CCL20	plasma at t0 IFN-γ	-0.12919	0.95	-0.51555	0.300829	2597.129	0.547423	24
serum at t0 CCL20	plasma at t0 IL-2	0.337248	0.95	-0.08912	0.65916	1524.33	0.107051	24
serum at t0 CCL20	plasma at t0 TNF-α	-0.33848	0.95	-0.65995	0.08774	3078.508	0.105692	24
serum at t0 CCL20	plasma at t0 IL-1β	-0.18317	0.95	-0.55502	0.24969	2721.291	0.391597	24
serum at t0 CCL20	plasma at t0 IL-6	-0.33616	0.95	-0.65847	0.090335	3073.177	0.108255	24
serum at t1 miR07	serum at t1 SERINC5	-0.13402	0.95	-0.51915	0.296352	2608.237	0.532417	24
serum at t1 miR07	serum at t1 IFN-β	0.458931	0.95	0.055556	0.733518	1244.458	0.024084	24
serum at t1 miR07	serum at t1 CCL20	-0.07071	0.95	-0.47086	0.353564	2462.641	0.742653	24
serum at t1 miR07	plasma at t0 CXCL10	0.305519	0.95	-0.1241	0.638679	1597.306	0.146555	24
serum at t1 miR07	plasma at t0 IFN-γ	0.038992	0.95	-0.38109	0.445727	2210.319	0.856454	24
serum at t1 miR07	plasma at t0 IL-2	0.013968	0.95	-0.40229	0.425437	2267.874	0.94835	24
serum at t1 miR07	plasma at t0 TNF-α	0.228559	0.95	-0.20474	0.586957	1774.315	0.28272	24
serum at t1 miR07	plasma at t0 IL-1β	-0.09074	0.95	-0.4864	0.335804	2508.696	0.673272	24
serum at t1 miR07	plasma at t0 IL-6	-0.01543	0.95	-0.42664	0.401059	2335.494	0.942944	24
serum at t1 SVRNA1	serum at t1 SERINC5	0.091089	0.95	-0.33549	0.486668	2090.496	0.672075	24
serum at t1 SVRNA1	serum at t1 IFN-β	0.020013	0.95	-0.39721	0.430376	2253.971	0.926049	24
serum at t1 SVRNA1	serum at t1 CCL20	0.240619	0.95	-0.19248	0.59526	1746.577	0.257384	24
serum at t1 SVRNA1	plasma at t0 CXCL10	-0.01657	0.95	-0.42757	0.400099	2338.122	0.938728	24
serum at t1 SVRNA1	plasma at t0 IFN-γ	0.088894	0.95	-0.33745	0.484978	2095.544	0.679562	24
serum at t1 SVRNA1	plasma at t0 IL-2	0.351062	0.95	-0.07356	0.667931	1492.557	0.092558	24
serum at t1 SVRNA1	plasma at t0 TNF-α	-0.08977	0.95	-0.48565	0.336668	2506.473	0.676567	24
serum at t1 SVRNA1	plasma at t0 IL-1β	-0.17161	0.95	-0.54671	0.260847	2694.706	0.422661	24
serum at t1 SVRNA1	plasma at t0 IL-6	-0.06581	0.95	-0.46702	0.357865	2451.37	0.759958	24
serum at t1 SVRNA2	serum at t1 SERINC5	0.067352	0.95	-0.35652	0.468227	2145.091	0.754511	24
serum at t1 SVRNA2	serum at t1 IFN-β	0.026026	0.95	-0.39213	0.435266	2240.14	0.903917	24
serum at t1 SVRNA2	serum at t1 CCL20	0.230417	0.95	-0.20286	0.588242	1770.04	0.278716	24
serum at t1 SVRNA2	plasma at t0 CXCL10	0.008005	0.95	-0.40727	0.42054	2281.589	0.970388	24

serum at t1 SVRNA2	plasma at t0 IFN-γ	0.102938	0.95	-0.32483	0.495744	2063.242	0.63219	24
serum at t1 SVRNA2	plasma at t0 IL-2	0.327116	0.95	-0.10041	0.652671	1547.634	0.118697	24
serum at t1 SVRNA2	plasma at t0 TNF-α	-0.11497	0.95	-0.50487	0.313895	2564.438	0.592677	24
serum at t1 SVRNA2	plasma at t0 IL-1β	-0.17168	0.95	-0.54675	0.260785	2694.854	0.422484	24
serum at t1 SVRNA2	plasma at t0 IL-6	-0.06469	0.95	-0.46614	0.358845	2448.793	0.76393	24
serum at t1 SERINC5	serum at t1 IFN-β	-0.04287	0.95	-0.44883	0.377765	2398.593	0.842354	24
serum at t1 SERINC5	serum at t1 CCL20	-0.0462	0.95	-0.45149	0.374902	2406.25	0.830279	24
serum at t1 SERINC5	plasma at t0 CXCL10	0.159341	0.95	-0.27257	0.537799	1933.516	0.457054	24
serum at t1 SERINC5	plasma at t0 IFN-γ	0.249108	0.95	-0.18377	0.60106	1727.052	0.240457	24
serum at t1 SERINC5	plasma at t0 IL-2	0.30808	0.95	-0.12132	0.64035	1591.415	0.143028	24
serum at t1 SERINC5	plasma at t0 TNF-α	0.221366	0.95	-0.21199	0.581969	1790.859	0.298553	24
serum at t1 SERINC5	plasma at t0 IL-1β	0.232434	0.95	-0.20082	0.589634	1765.401	0.274412	24
serum at t1 SERINC5	plasma at t0 IL-6	0.391499	0.95	-0.02677	0.693108	1399.553	0.058508	24
serum at t1 IFN-β	serum at t1 CCL20	0.124837	0.95	-0.30484	0.512294	2012.875	0.561101	24
serum at t1 IFN-β	plasma at t0 CXCL10	-0.07699	0.95	-0.47576	0.34803	2477.077	0.720666	24
serum at t1 IFN-β	plasma at t0 IFN-γ	-0.20318	0.95	-0.56923	0.230105	2767.305	0.340995	24
serum at t1 IFN-β	plasma at t0 IL-2	-0.03351	0.95	-0.44132	0.385771	2377.07	0.876476	24
serum at t1 IFN-β	plasma at t0 TNF-α	-0.34704	0.95	-0.66539	0.078108	3098.194	0.096616	24
serum at t1 IFN-β	plasma at t0 IL-1β	-0.20951	0.95	-0.57369	0.223828	2781.878	0.325822	24
serum at t1 IFN-β	plasma at t0 IL-6	-0.32557	0.95	-0.65167	0.102125	3048.803	0.120556	24
serum at t1 CCL20	plasma at t0 CXCL10	-0.04087	0.95	-0.44723	0.379479	2394	0.849615	24
serum at t1 CCL20	plasma at t0 IFN-γ	0.084801	0.95	-0.3411	0.481817	2104.958	0.693602	24
serum at t1 CCL20	plasma at t0 IL-2	0.140229	0.95	-0.29057	0.523756	1977.474	0.513404	24
serum at t1 CCL20	plasma at t0 TNF-α	-0.311	0.95	-0.64225	0.118132	3015.311	0.139076	24
serum at t1 CCL20	plasma at t0 IL-1β	-0.0553	0.95	-0.45873	0.367029	2427.194	0.797445	24
serum at t1 CCL20	plasma at t0 IL-6	-0.05355	0.95	-0.45734	0.36855	2423.161	0.803744	24
plasma at t0 CXCL10	plasma at t0 IFN-γ	0.764949	0.95	0.513664	0.895372	540.6175	1.34E-05	24
plasma at t0 CXCL10	plasma at t0 IL-2	0.28939	0.95	-0.14149	0.628083	1634.404	0.170191	24
plasma at t0 CXCL10	plasma at t0 TNF-α	0.244889	0.95	-0.18811	0.598182	1736.755	0.248775	24
plasma at t0 CXCL10	plasma at t0 IL-1β	-0.39074	0.95	-0.69264	0.027666	3198.697	0.059043	24
plasma at t0 CXCL10	plasma at t0 IL-6	0.335655	0.95	-0.0909	0.658144	1527.992	0.108823	24
plasma at t0 IFN-γ	plasma at t0 IL-2	0.103631	0.95	-0.3242	0.496272	2061.648	0.629887	24
plasma at t0 IFN-γ	plasma at t0 TNF-α	0.110507	0.95	-0.31797	0.501496	2045.834	0.607217	24
plasma at t0 IFN-γ	plasma at t0 IL-1β	-0.23032	0.95	-0.58818	0.202956	2829.742	0.278919	24

plasma at t0 IFN-γ	plasma at t0 IL-6	0.365992	0.95	-0.0565	0.677312	1458.219	0.078597	24
plasma at t0 IL-2	plasma at t0 TNF-α	-0.05004	0.95	-0.45455	0.371586	2415.091	0.816381	24
plasma at t0 IL-2	plasma at t0 IL-1β	-0.33523	0.95	-0.65787	0.091376	3071.035	0.109297	24
plasma at t0 IL-2	plasma at t0 IL-6	-0.05321	0.95	-0.45707	0.36884	2422.391	0.804947	24
plasma at t0 TNF-α	plasma at t0 IL-1β	0.303838	0.95	-0.12593	0.63758	1601.173	0.148903	24
plasma at t0 TNF-α	plasma at t0 IL-6	0.658755	0.95	0.336612	0.842856	784.8644	0.000465	24
plasma at t0 IL-1β	plasma at t0 IL-6	0.396695	0.95	-0.02062	0.696291	1387.601	0.054953	24

a T0, time at the first SARS-CoV-2 positive diagnosis. T1, time between 3 to 9 months after the first time. NPS, nasopharyngeal swab; CXCL-10/IP-10, Interferon-gamma Inducible Protein 10; IFN-γ, Interferon-gamma; IL-1 β, Interleukin-1β, IL-2, Interleukin-2; IL-6, Interleukin-6; TNF-α, Tumor Necrosis Factor-alpha; SERINC5, Serine incorporator protein 5; IFN-β, interferon beta; CCL20, chemokine ligand 20.
